# School-based hearing screening of first-grade students in Saudi Arabia: A pilot study

**DOI:** 10.4102/sajcd.v71i1.1063

**Published:** 2024-11-29

**Authors:** Noura I. Alothman, Ahmad A. Alanazi

**Affiliations:** 1 Department of Health Communication Sciences, College of Health and Rehabilitation Sciences, Princess Nourah bint Abdulrahman University, Riyadh, Saudi Arabia; 2 Department of Audiology and Speech Pathology, College of Applied Medical Sciences, King Saud bin Abdulaziz University for Health Sciences, Riyadh, Saudi Arabia; 3 King Abdullah International Medical Research Center, Riyadh, Saudi Arabia

**Keywords:** hearing loss, children, pure-tone audiometry, screening, school health service, Saudi Arabia

## Abstract

**Background:**

Hearing loss in children is a global concern. Early identification and intervention are critical for minimising the adverse effects of hearing loss. Despite the implementation of hearing screening programmes in Saudi Arabia, more research is needed on the audiological profiles of first-grade students.

**Objectives:**

This study aimed to characterize the audiological profile of first-grade students in Saudi Arabia and to raise awareness among parents, schools, and decision-makers about the need for hearing screening programs and the implementation of an effective monitoring and management system in the country.

**Method:**

This cross-sectional descriptive study assessed the audiological profile of 308 first-grade students in Riyadh, the capital of Saudi Arabia, using pure-tone audiometry (PTA) and tympanometry. Additionally, two questionnaire-based assessments, one for parents and one for the home teacher, were developed to predict students who failed PTA.

**Results:**

Among the students screened, 26.3% failed PTA. Tympanometry identified abnormal middle ear function in 30.5% of the students. The discrimination capacity of both questionnaires in identifying students who failed PTA screening was poor.

**Conclusion:**

A 26.3% prevalence rate of PTA screening failure, and a 30.5% of middle ear pathologies were noted. Both parent and teacher questionnaires were ineffective tools for identifying hearing loss among first-grade children who failed PTA screening.

**Contribution:**

This pilot study recommends that primary schools incorporate hearing screening as a regular practice of their preventative healthcare system. The study emphasises the importance of establishing international standards for school-based hearing screening to enhance its efficacy and develop more effective hearing screening questionnaires.

## Introduction

According to the World Health Organization (WHO), 34 million children are affected by hearing loss (WHO, 2024). Hearing disability is defined as a hearing threshold in the better-hearing ear that exceeds 35 decibels hearing level (dB HL) (WHO, 2024). The impact of hearing loss on a child’s speech and language development has been well documented (Lieu et al., 2020; Pimperton & Kennedy, 2012; Shukla et al., 2020). Children with untreated hearing loss often experience delays in the development of speech, language, social skills, cognitive abilities and learning (Shojaei et al., 2016; Shukla et al., 2020). Moreover, the academic achievement levels of children with untreated hearing loss have been reported to be lower than those of their peers with normal hearing (Foster et al., 2023). Even a slight degree of hearing loss can negatively affect a child’s life. For instance, children with hearing loss exceeding 26 dB HL difficulties in comprehending soft speech, especially in noisy environments or from a distance (WHO, 2024).

Negative consequences of hearing loss can be minimised or prevented through early identification and intervention (Joint Committee on Infant Hearing [JCIH], 2007; Kennedy et al., 2005). Implementing hearing screening programmes for different age groups is crucial for early identification and intervention (JCIH, 2007; WHO, 2010). Yong et al. (2020) reviewed existing school hearing screening programmes around the world and identified the absence of studies indicating the actual prevalence of hearing loss in school-age children. While prevalence estimates ranged from 0.9% in Taiwan to 34.0% in Brazil (Nogueira & Mendoncxa, 2011; Yang et al., 2011), several additional studies gave referral percentages for screening tests instead of prevalence (Yong et al., 2020). In Saudi Arabia, hearing screening programmes were introduced for newborns and school-aged children in 2016 and 2018, respectively (Ministry of Health, 2016, 2019). All newborns in the country, across all birth hospitals, undergo screening through the newborn hearing screening programme, while all first-grade students in schools throughout the country are involved in the school hearing screening programme. The Ministry of Health bears primary responsibility for implementing these programs nationwide.

Although the school-hearing programme in Saudi Arabia was launched in 2018, only a few studies have reported its outcomes. After the first academic year of implementing school hearing screening, the Ministry of Health reported a hearing loss prevalence of 11% among first-grade students during the academic year 2018–2019 (Ministry of Health, 2019). However, specific information regarding the screening methodology employed is lacking, and it is unclear whether all students underwent screening or only those suspected of having hearing loss were included. On the other hand, Al Daajani et al. (2021) reported a hearing loss prevalence of 0.7% among 15 426 first-grade students during the same academic year, 2018–2019 (Al Daajani et al., 2021). However, their study included only students suspected of having hearing difficulties by their teachers, introducing a potential limitation based on individual teacher judgement and experience. Therefore, the prevalence seemingly differs from that reported in studies conducted before the launch of school hearing screening in Saudi Arabia.

Al-Rowaily et al. (2012) assessed the hearing of 2,574 children, aged 4–8 years, before kindergarten and primary school entry in Riyadh, the capital of Saudi Arabia, and reported a hearing loss prevalence of 1.75%, with conductive hearing loss being the primary type. Alharbi and Ahmed (2015) tested 1220 kindergarten children aged 4–6 years in Jazan, a city in Saudi Arabia, and reported a hearing loss prevalence of 3.10%, mainly because of upper respiratory infections or secretory otitis media. Thus, research on the audiological profiles of first-grade students in Saudi Arabia is lacking. Despite several attempts to obtain permission to access the data registry of the national school hearing screening programme, such requests have been consistently denied. Consequently, this study was designed to address this gap in knowledge by investigating the hearing status of grade-one students to raise further awareness among parents, schools and decision makers (e.g., the Ministry of Health and the Ministry of Education) about the need for hearing screening programmes and the implementation of an effective monitoring and management system in Saudi Arabia.

## Research methods and design

This cross-sectional descriptive study was designed to assess the audiological profile of first-grade students in Riyadh, Saudi Arabia. First-grade students aged 6–7 years from eight schools in Riyadh were invited to participate in this study. Only the authors had access to the data from this study.

### Hearing screening

An otoscopic examination was conducted for each student. The functionality of the outer and middle ears was assessed using a portable tympanometer (MT10; Intracoustics, Middelfart, Denmark). Pure-tone audiometry (PTA) screening was performed using a calibrated portable audiometer (AD-226; Interacoustics, Middelfart, Denmark) with a TDH39 dd45 audiometric headset for an air-conducted pure tone at frequencies of 0.5, 1, 2, 4 and 8 kHz at 20 dB HL. Regarding the frequencies utilised in hearing screening, numerous recommendations and guidelines are in place. Because of concerns about pass or fail rates, one guideline, for instance, recommends excluding 0.5 kHz and screening at the three frequencies of 1, 2 and 4 kHz alone (American Speech-Language-Hearing Association, 1997). For low frequency diseases (e.g., otitis media), another suggests adding 0.5 kHz (American Academy of Audiology, 2011). Several organisations mandate different combinations of screening frequencies, and some suggest including 6 kHz for noise-induced hearing loss (Meinke & Dice, 2007). A hearing screening threshold of 25 dB HL may be used if ambient noise is loud (Bamford et al., 2007; Mahomed-Asmail et al., 2016).

Students responded by verbalising ‘yes’ or raising their hands. Each student was required to respond to all frequencies in both ears to pass the PTA screening. Testing was performed by certified audiologists on the same day for each student in a quiet room away from known noise sources at the school. School principals were informed about the results of students who failed the screening and the necessity of rescreening these cases.

### Questionnaire

Two questionnaires, one for parents and the other for the home teachers, were initially developed in English, translated into Arabic, and administered for data collection after a literature review and a focus group of experts in the field. The parents’ questionnaire included eight questions that explored their perspective on the child’s potential hearing impairment, frequency of asking for repetitions, requesting the speaker to raise their voice, speaking volume, understanding of instructions without looking, sentence length compared with their peers, linguistic vocabulary and whether anyone suggested that the child might have a hearing impairment. The home teacher’s questionnaire comprised of nine questions investigating the students’ academic level, responsiveness to the teacher’s questions, belief in possible hearing impairment, frequency of asking for repetitions, requests for the speaker to raise their voice, speaking volume, understanding of instructions without looking, sentence length compared with their peers and the child’s linguistic vocabulary compared with his or her peers.

Because Arabic is the official language of Saudi Arabia, the authors followed the WHO guidelines for the translation and adaptation of the instruments (WHO, 2012). Two independent bilingual experts (in English and Arabic) translated the original English questionnaire into Arabic. The experts then recognised and addressed any insufficient translational phrases or ideas. The Arabic version was translated back into English by two independent bilingual experts. No changes were made to the translated versions, which were considered the final versions of the questionnaire. The population size of parents and teachers who agreed to fill out both questionnaires was small; therefore, piloting the questionnaires for solely validation purposes was not performed to avoid excluding the participants from the actual study. Consequently, the reliability of the questionnaires could not be determined using statistical techniques such as Cronbach’s alpha or inter-rater reliability.

### Statistical analysis

The results were recorded using a standardised recording form for screening data and analysed descriptively and numerically. The percentages of passing or failing the PTA screening and normal or abnormal tympanograms were calculated. Responses from parents’ and teachers’ questionnaires were compared with PTA screening results to determine any association using Spearman correlation, owing to the non-normal distribution of the data (Kolmogorov–Smirnov test, *p* ≤ 0.05). Statistical significance was set at *p ≤* 0.05.

The effectiveness of each questionnaire in predicting the PTA screening results was assessed using receiver operating characteristic (ROC) curve analysis. The area under the ROC curve, which represents the overall performance of the questionnaire, was calculated, with higher values indicating better discrimination between true- and false-positive results. Additionally, the optimal cut-off value was determined by identifying the point on the ROC curve that was closest to the upper-left corner of the unit square, where both sensitivity and specificity were equal to one, signifying a perfect test (Akobeng, 2008; Perkins & Schisterman, 2006). All statistical analyses were performed using IBM SPSS (Statistical Package for the Social Sciences) Statistics for Windows version 20 (IBM Corp., Armonk, New York, United States).

### Ethical consideration

Ethical approval was obtained from the Princess Nourah bint Abdulrahman University Institutional Review Board (no: 22-0623). Parents received an informed consent form containing comprehensive details about the study electronically, and enrolment was exclusive to those who explicitly agreed to participate.

## Results

### Hearing screening

Overall, 308 students including 226 girls (73%) and 82 boys (27%) were enrolled in the analysis. All students were screened for hearing via the PTA, resulting in a total of 616 ears being tested. Tympanometry was conducted in 305 students, excluding three boys owing to hard wax blockage and atresia (*n* = 2) and microtia (*n* = 1), resulting in 610 ears being tested.

**FIGURE 1:**
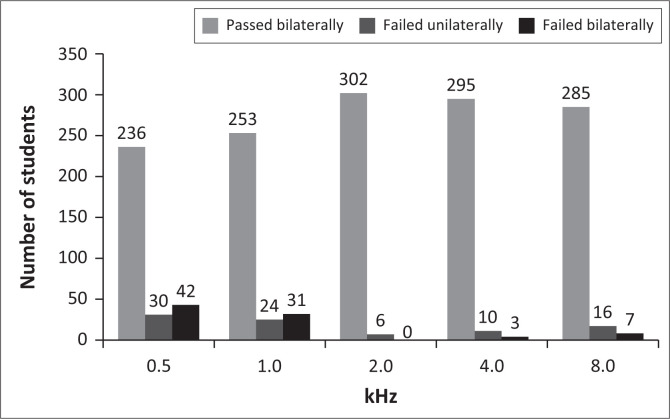
Number of students who passed or failed pure-tone audiometry screening at different frequencies.

The test results indicated that 73.7% of the students (227 girls and 56 boys) passed the PTA screening (Table 1). The remaining 26.3% (55 girls and 26 boys) failed bilateral PTA screening. More than half of the students who failed the PTA screening failed at 0.5 and/or 1 kHz. Approximately 87.8% of the students who failed the PTA screening (*n* = 71) failed in either one or both ears at 0.5 kHz, followed by 1 kHz (67%), 8 kHz (39%), 4 kHz (15%) and 2 kHz (7%) (Figure 1).

**TABLE 1:** Results of pure-tone audiometry screening (pass versus fail) and tympanometry (normal versus abnormal).

Variable1	Total		Girls		Boys	
	*n*	%	*n*	%	*n*	%
**PTA screening**	308	100.0	226	73.0	82	27.0
Pass	227	73.7	171	75.6	56	68.3
Fail	81	26.3	55	24.3	26	31.7
**Tympanometry**	305	100.0	226	74.1	79	25.9
Normal bilaterally	212	69.5	155	68.6	57	72.2
Normal unilaterally	41	13.4	34	15.0	7	8.9
Abnormal bilaterally	52	17.1	37	16.4	15	18.9

PTA, pure-tone audiometry.

Tympanometry results showed that 69.5% of the students (*n* = 212) had normal bilateral middle ear function (type A). The remaining students had abnormal middle ear function, either bilaterally (17.1%) or unilaterally (13.4%). The most frequent tympanogram types among students with abnormal middle ear function were As and C, which were found in 53 and 42 ears, respectively (Table 2). Notably, 46.9% of the students who failed the PTA screening also had abnormal middle ear function.

**TABLE 2:** Type of tympanometry in students with abnormal middle ear function (*n* = 71 girls, *n* = 22 boys).

Variable	Type B		Type C		Type As		Type Ad		Combination†	
	*n*	%	*n*	%	*n*	%	*n*	%	*n*	%
**Bilateral**										
Girls	7	9.8	6	8.5	15	21.2	1	1.4	8	11.3
Boys	2	9.2	10	45.5	0	0.0	0	-	3	13.6

Total	9	9.6	16	17.3	15	16.2	1	1.1	11	11.8

**Unilateral**										
Girls	7	9.8	7	9.8	16	22.6	4	5.6	-	-
Boys	0	0.0	3	13.6	1	4.5	3	13.6	-	-

Total	7	7.5	10	10.7	17	18.3	7	7.5	-	-

† , Combination: two different types of tympanograms for both ears.

### Questionnaire

Teacher responses were received for 150 students who passed the screening (total = 227 students) and 39 students who failed the screening (total = 81 students). Two groups of equal numbers of students were selected to assess the association between the teachers’ responses and PTA screening results. Groups 1 and 2 included teachers’ responses for 39 students who failed the screening and for 39 students randomly selected from among 150 passing students, respectively. Parents’ responses for the same students were also analysed.

The responses of parents and teachers to the questionnaire are shown in Table 3 and Table 4, respectively. Regarding parents’ responses to the questionnaire, significant correlations were found between the PTA screening results and responses to the question ‘Does your child speak relatively loudly?’ (*r* = 0.23) and ‘Does your child have a limited linguistic vocabulary compared with his or her peers?’ (*r* = 0.34). Furthermore, there was a significant correlation between PTA screening results and teachers’ responses to the question, ‘Does your child speak relatively loudly?’ (*r* = 0.25). No other significant correlations were found between the PTA screening results and responses to other questions in the questionnaires. Interestingly, most parents believed that their children did not speak loudly and did not have a limited vocabulary, regardless of whether their children passed or failed the PTA screening. Similarly, teachers’ responses indicated that most students did not speak loudly.

**TABLE 3:** Correlation between pure-tone audiometry screening results and parents’ responses to the questionnaire (*N* = 39 in each group).

Questions	PTA		*p*
	Pass	Fail	
Do you believe that the child has a hearing impairment?			CBT
Yes	0	0	-
No	39	39	-
Does the child ask you to repeat what is said to him or her more than once?			> 0.05
Yes	2	1	-
No	37	38	-
Does the child ask the speaker to raise their voice so that he or she can understand what is being said to him or her?			> 0.05
Yes	1	0	-
No	38	39	-
Does the child speak relatively loudly?			0.03*
Yes	4	0	-
No	35	39	-
Does the child understand what is asked of him or her without looking at the speaker?			> 0.05
Yes	22	28	-
No	17	11	-
Does the child speak in short sentences compared with his or her peers?			> 0.05
Yes	10	11	-
No	29	28	-
Does the child have limited linguistic vocabulary compared with his or her peers?			0.005*
Yes	9	1	-
No	30	38	-
Has anyone mentioned to you that your child may have a hearing impairment?			> 0.05
Yes	1	1	-
No	38	38	-

PTA, pure-tone audiometry; CBT, cannot be tested.

* , Statistically significant.

**TABLE 4:** Correlation between pure-tone audiometry screening results and teachers’ responses to the questionnaire (*N* = 39 in each group).

Questions	PTA		*p*
	Pass	Fail	
What is the academic level of the child?			> 0.05
Good	39	38	-
Bad	0	1	-
Does the child respond to the teacher’s questions?			> 0.05
Yes	33	35	-
No	6	4	-
Do you believe that the child has a hearing impairment?			> 0.05
Yes	2	4	-
No	37	35	-
Does the child ask you to repeat what is said to him or her more than once?			> 0.05
Yes	0	2	-
No	39	37	-
Does the child ask the speaker to raise their voice so that he or she can understand what is being said to him or her?			> 0.05
Yes	2	1	-
No	37	38	-
Does the child speak relatively loudly?			0.025*
Yes	7	1	-
No	32	38	-
Does the child understand what is asked of him or her without looking at the speaker?			0.06
Yes	17	26	-
No	22	13	-
Does the child speak in short sentences compared with his or her peers?			> 0.05
Yes	12	12	-
No	27	27	-
Does the child have a limited linguistic vocabulary compared with his or her peers?			> 0.05
Yes	8	9	-
No	31	30	-

PTA, pure-tone audiometry.

* , Statistically significant.

**FIGURE 2:**
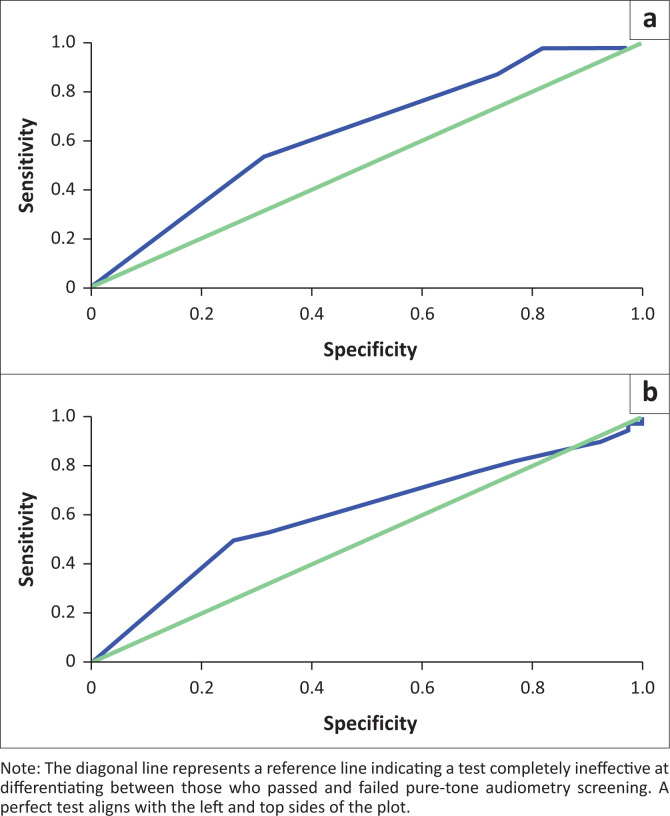
Receiving operator characteristic curves for parents’ (a) and teachers’ (b) questionnaires predicting the outcomes of pure-tone audiometry screening.

The results of the ROC analysis are shown in Figure 2, which indicates that the area under the ROC curve was 0.64 for the parents’ questionnaire (95% confidence interval [CI]: 0.51–0.76) and 0.60 for the teacher’s questionnaire (95% CI: 0.48–0.73), indicating that both questionnaires have poor discrimination capacity to distinguish between children who passed and failed PTA screening. Regarding the parents’ questionnaire, the optimal cutoff value for predicting the outcomes of the PTA screening was found to be 11.5 points with a sensitivity of 0.54 and specificity of 0.32. The cutoff value for the teacher’s questionnaire was 17.5, with a sensitivity of 0.53 and a specificity of 0.24.

## Discussion

The results of this pilot study revealed a hearing loss prevalence of 26.3%, which is higher than that reported previously, among school-aged students in Saudi Arabia. Earlier studies reported hearing loss prevalence rates of 1.75%, 3.10% and 0.7% (Al Daajani et al., 2021; Alharbi & Ahmed, 2015; Al-Rowaily et al., 2012). These variations in the reported prevalence could be attributed to differences in methodologies for determining hearing loss across studies, ranging from confirmed hearing loss with diagnostic audiometry (Alharbi & Ahmed, 2015; Al-Rowaily et al., 2012) to screening failure rates, as in the current study and the study conducted by Al Daajani et al. (2021). Despite the reliance of our study and that of Al Daajani et al. (2021) on screening outcomes for prevalence data, the higher prevalence in the current study may be attributed to several factors. Firstly, the present study included all first-grade students, in contrast to the study by Al Daajani et al. (2021), which involved only students suspected of having potential hearing difficulties by their teachers based on individual teacher judgement and experience. Secondly, the screening level used in the current study, with a pass criterion of 20 dB HL, differed from the 25 dB HL criterion used by Al Daajani et al. (2021). Thirdly, the inclusion of 0.5 kHz in the current study’s screening protocol contributed to a higher failure rate, given that 0.5 kHz is more susceptible to chronic noise interference and middle ear pathologies (Kim & Koo, 2015; Yilmaz et al., 2022). Additionally, including 8 kHz in the current study’s protocol could further contribute to the higher prevalence reported in the current study, given that 8 kHz is more susceptible to louder sounds or noises (Owens, 2008). Notably, in the current study, 87.8% and 39.0% of the screening failures occurred at 0.5 kHz and 8 kHz, respectively. Fourthly, the screening in the current study was conducted during winter, which may have contributed to the high number of students with abnormal middle ear function (30.5%). Finally, the smaller sample size in this study compared to that of Al Daajani et al. (2021) may also have contributed to the reported differences in prevalence rates between the studies.

In comparison to other countries, the prevalence of hearing loss among school-aged children was 18.3% – 34.0% in African countries (Skarżyński et al., 2014) and 15.9% – 24.1% in Asian countries (Skarżyński et al., 2020). Moreover, it was found that 11.9% of Indian children, 20.5% of Polish children and 10.0% of Iranian children aged 7 years to 8 years had hearing loss (Ross et al., 2008; Sarafraz & Ahmadi, 2009; Swierniak et al., 2021). The differences in screening protocols may be owing to the absence of international guidelines for school hearing screening (Yong et al, 2020). Therefore, standardised school screening programmes worldwide must be established to conduct higher-quality studies that precisely estimate region-specific hearing loss prevalence and assist in the creation of guidelines to maximise screening test sensitivity and specificity (Yong et al, 2020).

In the current study, a prevalence rate of 30.5% for middle ear pathologies was observed. It is worth noting that such issues are particularly prevalent in younger children, especially during winter when the screening was conducted. In a previous study, 84.4% of school-aged children with hearing loss also had middle ear problems (Al-Rowaily et al., 2012). Among the students who failed screening in our study, 46.9% had middle ear pathologies. The most common tympanogram types among the participating students were types As and C. Type As suggests reduced compliance and potential stiffness in the middle ear, while type C indicates negative pressure in the middle ear, signifying Eustachian tube dysfunction. Previous studies have highlighted the prevalence of otitis media in school-aged children (Alharbi & Ahmed, 2015; Al-Rowaily et al., 2012). The stiffness observed in 34.5% of the students with middle ear pathologies might be linked to otitis media. Remarkably, all children with Type As were girls, except for one boy, suggesting a potential link to otosclerosis. It has been reported that young women are particularly susceptible to otosclerosis (Ricci et al., 2022). Regarding a type C tympanogram, the complex relationship between Eustachian tube dysfunction and otitis media, where each influences the other, has been documented (Rosenfeld, 2005; Teele et al., 1980). Alharbi and Ahmed (2015) identified upper respiratory infections as the leading cause of middle ear pathology in children aged 4–6 years. The prevalence of type C tympanograms in the current study could be linked to upper respiratory infections affecting the Eustachian tube. Further tests are necessary for a conclusive diagnosis of middle ear pathologies, including the underlying causes, among students.

In addition to PTA and tympanometry, subjective measurements such as those performed using questionnaires can also be used to identify hearing loss in school-aged children (Bamford et al., 2007; WHO, 2012). Questionnaire-based screening has been developed and implemented because it is inexpensive and can be used to screen large numbers of children in less time, without the need for training test personnel (Muñoz et al., 2014; Newton et al., 2001). Muñoz et al. (2014) reviewed the literature on the efficacy of using parent- or teacher-completed questionnaires to screen school-aged children for hearing loss. The authors concluded that there was insufficient evidence to support the sensitivity and specificity of these questionnaires as screening tools. Questionnaires can only alert parents or teachers about bilateral or more severe hearing loss. They cannot effectively help identify unilateral or mild hearing loss based only on observation of behaviours or responses to questions (Muñoz et al., 2014). The current study found that parent and teacher questionnaires were ineffective tools, with low sensitivity and specificity for identifying hearing loss among first-grade children who failed PTA screening.

Several factors may have contributed to the poor performance of the questionnaires. Firstly, despite being developed based on literature reviews and expert inputs, the questions may not have comprehensively covered the full range of symptoms or behaviours associated with hearing loss. Secondly, the subjective interpretation of the questions by parents and teachers may have led to inaccurate responses. For instance, most children who failed PTA screening were reported not to speak loudly by their parents and teachers. However, it is well-documented that children with hearing loss often speak loudly to compensate for difficulties in understanding or being heard clearly (Yigider et al., 2020). The perception of speaking loudly may vary among individuals. Moreover, administering such questionnaires without prior training to parents and teachers may have contributed to inaccurate responses. Insufficient training on interpreting the questions could have further lowered the sensitivity and specificity of the questionnaires.

Addressing these issues through an improved questionnaire design and standardised training for a larger sample size of parents and teachers may enhance the effectiveness of these questionnaires as screening tools for hearing loss in school-aged children. Despite the authors’ intensive efforts to recruit the required number of participants, the small sample size was the primary limitation. Another limitation was that diagnostic evaluations of students who failed the PTA screening were not conducted.

## Conclusion

School-based hearing screening helps identify children with hearing loss who require further services to reduce the negative consequences of hearing loss. School-based hearing screening is an essential element of health education for parents and teachers. This pilot study addressed the audiological profiles of first-grade students in Riyadh. Differences in the screening protocols used in similar studies conducted in Saudi Arabia and worldwide may lead to different results in hearing screening. The findings of this pilot study highlighted the need to establish unified international guidelines for school hearing screening to enhance the efficacy of these programmes worldwide. Questionnaire-based hearing screening can be helpful when sensitive and specific instruments are used. Therefore, there is a need to develop more effective hearing screening questionnaires.
